# Utility of different cardiovascular disease prediction models in rheumatoid arthritis

**Published:** 2014

**Authors:** A Purcarea, S Sovaila, G Udrea, E Rezus, A Gheorghe, C Tiu, V Stoica

**Affiliations:** * Strasbourg Medical University, Civil Hospital Strasbourg, Internal Medicine Department, France; ** Civil Hospital Strasbourg, Internal Medicine Department, France; *** Geomed-Klinik Hospital, Gerolzhofen, Internal Medicine Department, Germany; **** ”Carol Davila” University of Medicine and Pharmacy, “Cantacuzino” Hospital, Internal Medicine and Rheumatology Department, Bucharest, Romania; ***** ”Carol Davila” University of Medicine and Pharmacy, University Emergency Hospital, Neurology Department, Bucharest, Romania; ****** ”Grigore T. Popa” University of Medicine and Pharmacy, Rheumatology Department, Iasi, Romania

**Keywords:** cardiovascular disease prediction models, rheumatoid arthritis, risk assessment, SCORE, SCORE, carotid intima media

## Abstract

**Background.** Rheumatoid arthritis comes with a 30% higher probability for cardiovascular disease than the general population. Current guidelines advocate for early and aggressive primary prevention and treatment of risk factors in high-risk populations but this excess risk is under-addressed in RA in real life. This is mainly due to difficulties met in the correct risk evaluation. This study aims to underline the differences in results of the main cardiovascular risk screening models in the real life rheumatoid arthritis population.

**Methods.** In a cross-sectional study, patients addressed to a tertiary care center in Romania for an biannual follow-up of rheumatoid arthritis and the ones who were considered free of any cardiovascular disease were assessed for subclinical atherosclerosis. Clinical, biological and carotidal ultrasound evaluations were performed. A number of cardiovascular disease prediction scores were performed and differences between tests were noted in regard to subclinical atherosclerosis as defined by the existence of carotid intima media thickness over 0,9 mm or carotid plaque.

**Results.** In a population of 29 Romanian rheumatoid arthritis patients free of cardiovascular disease, the performance of Framingham Risk Score, HeartSCORE, ARIC cardiovascular disease prediction score, Reynolds Risk Score, PROCAM risk score and Qrisk2 score were compared. All the scores under-diagnosed subclinical atherosclerosis. With an AUROC of 0,792, the SCORE model was the only one that could partially stratify patients in low, intermediate and high-risk categories. The use of the EULAR recommended modifier did not help to reclassify patients.

**Conclusion.** The only score that showed a statistically significant prediction capacity for subclinical atherosclerosis in a Romanian rheumatoid arthritis population was SCORE. The additional calibration or the use of imaging techniques in CVD risk prediction for the intermediate risk category might be warranted.

## Introduction

Cardiovascular disease (CVD) is responsible for a 30%-50% mortality excess in rheumatoid arthritis (RA) patients [**1**]. Similar to the general population, intensified measures to control the risk factor are recommended in high-risk patients [**2**,**3**]. This relies on an accurate capacity to predict this CVD risk which itself reposes on the use of the two guideline-recommended [**2**] clinical scores. These recommendations are based on data with a low level of evidence and their impact in real life is not yet well known.

SCORE [**4**] is the first of the two models recommended both by the European Cardiology Society [**5**] (ESC) and The European League against Rheumatism [**2**] (EULAR) for the estimation of the 10 year CVD mortality in the general population and rheumatoid arthritis. It is derived from pooled European cohorts and modifiers apply to the geographical diversity. In order to comply with the proven excess mortality in RA, EULAR advocates for the use of a multiplier adapted to the disease’s specific conditions resulting in a new SCORE: the mSCORE. SCORE BMI is an office-based tool derived from the same model, which replaces the lipid profile with the more office-friendly body mass index (BMI). The second score recommended by EULAR is the Framingham risk score (FRS) [**6**], using the same multiplier for RA patients. It is the best-known CVD prediction model and it derives from an US cohort, FRS. It is not specifically customized for the European populations and it measures both fatal and nonfatal CVD events. Several other risk scores are available: PROCAM [**7**] is derived from a German cohort and it is tested as CVD risk prediction. QRisk2 [**8**] and its office based alternative (QRisk2 - BMI) are models designed in UK as a multi-ethnic tool for the 10y prediction of the CVD events. It has the particularity to include RA as an independent risk factor and to adapt to social deprivation as another risk factor. The Reynolds Risk Score (RRS) [**9**] is derived from an US cohort by a team of pioneers of the inflammatory hypothesis in atherosclerosis and uses hs CRP as a risk factor. The ARIC model is based on another US cohort and it is based on the same traditional risk factors. It has a variant using the carotid intima media thickness as a variable [**10**]. The ARIC, PROCAM, Qrisk, RRS and FRS were not calibrated for a wider European population.

Subclinical atherosclerosis, a predictor of CVD events, can be assessed with ultrasound techniques. Carotid intima media thickness (cIMT) is widely used as an independent surrogate marker of subclinical atherosclerosis and cardiovascular events [**11**,**12**] and in RA it seems to have an advantage over the other imaging techniques [**13**].

## Objectives

To evaluate the advantages, limits and differences of several models for CVD risk assessment in Romanian rheumatoid arthritis population, free of cardiovascular disease.

## Methods

A cross sectional study conducted on over 6 months, between June 2008 and January 2009 in the Rheumatology Department of “Dr. I. Cantacuzino” Hospital and the Neurology Department of the Emergency University Hospital, both affiliated to “Carol Davila” University of Medicine and Pharmacy, Bucharest. The study represents the initial cross-sectional phase of a longitudinal project and was approved by the Ethical Committees of both hospitals and “Carol Davila” University of Medicine and Pharmacy, Bucharest. An informed consent was obtained from all the subjects enrolled in the study.

**Patient population:** A number of consecutive RA patients free of previous cardiovascular disease who were addressed for their bi-annual evaluation were enrolled in the Rheumatology clinic.

**Laboratory tests:** Serum levels of total cholesterol, triglycerides, HDL cholesterol and creatinine were measured by enzymatic methods adapted to an auto-analyzer (Cormay Accent 300). Renal function was estimated with the MDRD formula. Lipid categories are defined by the ATP III / ESC guidelines [**14**].

**Ultrasound measurements (US):** CIMT was measured by using the CIMT imaging protocol [**15**] with the patient supine by the same trained assessor. It represented the mean of 3 bilateral measurement values of the intima-media thickness of the near and far common carotid artery wall. The measurement was performed at 1 centimeter before the carotid bifurcation. IMT represents the cumulated thickness of the intima (echogenic) and media (echolucent). Plaque is defined as a luminal protruding focal structure of at least 0.5 mm or 50% of the surrounding IMT value or a thickening of more than 1,5 mm of the carotid artery, in conformity with the Mannheim consensus [**16**] at any focal location in the extra cranial carotid arteries. Measurements were realized with a 7.5 MHz Linear probe (Siemens Sonoline).

**Gold standard:** subclinical atherosclerosis as defined by a mean cIMT greater than 0,9mm or the existence of focal plaque [**15**,**17**,**18**].

**Predictive scores:** Framingham risk score (FRS) and EULAR modified FRS (mFRS [**2**]), HearSCORE (SCORE) and EULAR modified SCORE (mSCORE [**2**]), Qrisk2 score (Qrisk2), PROCAM score (PROCAM), ARIC score (ARIC) were calculated with the official tools provided by the authors. When age limits were crossed, the actual age was substituted with the minimal/ maximal ages accepted by the official tool.

Statistical analysis: Data was verified for normality with the Shapiro test and treated accordingly and was presented as median (range) or mean (IC95). Statistical significant variance between groups was calculated with the Mann-Whitney U. The strength of correlation with the primary outcome was assessed by using the Spearman’s Rho correlation coefficient and the chi square test was used to estimate the likelihood ratios. An agreement between the effective score results was assessed with the intra class coefficient (ICC). ICC values over .6 were considered statistically significant, values over .8 were considered good and over .9 were considered excellent. Goodman–Kruskal γ test was used to measure the strength of association at the ordinal levels. The statistical analysis was performed by using SPSS for Windows release 22, Chicago, Illinois, and p<.05 was considered significant.

## Results

**Main characteristics of the population:**

29 rheumatoid arthritis patients with a mean age of 54.9 (in 2009) were included. They were mainly women (28/29). The mean disease duration was of 10,5 years (1-35) and the disease activity (DAS28- VS) was 5,12 (1,86-6,54). They were all treated with DMARDs (29/29) either alone or in combinations and 5 (17%) received a biological TNF blocker. Methotrexate was used in 26 patients (90%) alone or in association. Glucocorticoids were used at the moment of the census in 7 (24%) out of 29 patients and NSAID, which was used more than occasional, was reported in 9 patients (31%). Extra-articular disease was present in 3 patients (10%). The mean cardiovascular CVD risk factors prevalence was of 2,48 (0-5). One patient smoked. 4 (14%) had a relevant CVD family history. Obesity was present in 9 (31%) patients with a mean BMI of 27.2. High LDL cholesterol was present in 14 patients (48%). The total cholesterol was M= 200mg/dl (136-2981) with an HDL cholesterol of M= 52 mg/dl (25-98). Diabetes was present in 15 patients (51%), hypertension in 11 patients (38%) and chronic kidney disease in 4 patients (14%). Except for ESR, there were no statistically significant differences between the patients with subclinical atherosclerosis and those without.

Additional data are presented in **Table 1**.

**Table 1:** Characteristics of the population

	Overall Mean	Range	Subclinical atherosclerosis present	Subclinical atherosclerosis absent	P Value ǂ
Age (years)	54,93	(34 - 76)	61,6	52	,757
Systolic blood pressure	129,83	(110 - 160)	133	127	,322
Disease duration (years)	10,5862	(1,00 - 35,00)	10,2	11,3	,275
ESR (mm/h)	33,17	(1 - 104)	40,3	26,5	,025*
DAS28 - VS score	5,12	(1,86 - 6,54)	4,98	5,3	,293
BMI	27,2452	(20,81 - 36,33)	26,46	27,78	,535
hs - CRP	33,23838	(12,731 - 269,914)	13,9	17,8	,959
Total Cholesterol (mg/l)	199,55	(136 - 281)	191	204	,645
HDL Cholesterol (mg/l)	52,48	(25 - 98)	48	52	,595
LDL Cholesterol	112,45	(46 - 294)	127	130	,085
GFR estimate (CKD - EPI) ml/min	78,1379	(57,00 - 103,00)	76	80	,574
Mean risk factors	2,48	(0 -5)	3	2,6	,909
ǂ *Man-whitney U for equality of variance*

**IMT measurement results**

Subclinical atherosclerosis was diagnosed by using the ultrasound carotid evaluation in 12 patients (41,4). Of these, focal plaque was present in 9 (31%) of the 29 patients and a mean cIMT ≥ 0,9mm was found in 7 patients (24%).

**Clinical scores overall results**

Cardiovascular disease risk classes were calculated by using 11 prediction models. Of those, 8 were mixed clinical and laboratory models and 3 were office based models substituting the lipid profile with the BMI. The main results are available in **Table 2**, **3** and **4**.

**Table 2:** Overall CVD risk score results

	Mean	range	Subclinical atherosclerosis present: mean (95%IC)	Subclinical atherosclerosis absent mean (95%IC)	P value ǂ
SCORE	1,41	0-5	2,08(1,34-2,82)	1(0,27-1,72)	,011*
SCORE BMI	2,79	0-9	3,75(2,28-5,21)	2,25(0,9-3,56)	,082
mSCORE	1,55	0-7,5	2,04 (1,4-2,6)	1,28 (0,2-2,3)	,02*
mSCORE BMI	3,1	0-9	4,16(2,66-5,66)	2,65 (1,04-4,22)	,082
FRS	12,03	1,4-31,3	13,67(9,42-17,92)	11,38(7,2-15,54)	,324
mFRS	13,97	31,3	15,55(10,9-20,18)	13,49(8,54-14,44)	,324
Qrisk2	13,95	0,5-37,7	17,17 (10,7-23,64)	12,2(6,2-18,2)	,110
Qrisk2 - BMI	13,48	0,6-35,6	16,52 (9,57-23,4)	11,65(5,8-17,48)	,159
PROCAM	8,5	0-62,2	9,5 (1,98-17,03)	8,15(0,06-16.29)	,233
RRS	4,03	1-17	5,33 (2,32-8,34)	3,25(1,93-4,56)	,189
ARIC	7,01	0,12-19,7	6,75 (4,14-9,35)	7,65(4,63-10,66)	,945
ǂ *Man-whitney U for equality of variance*

**Table 3:** Crosstab risk classification between the gold standard and the predictive models

**Predictive model**	**Risk category**	**Subclinical atherosclerosis present**	**Subclinical atherosclerosis absent**
FRS	Low	69% (0/4)	31% (4/4)
	Intermediate low and high	38% (10/20)	62% (10/20)
	High and very high	62% (2/5)	38% (3/5)
mFRS	Low	69%(1/5)	31% (4/5)
	Intermediate low and high	37.5% (3/16)	62.5% (5/16)
	High and very high	62.5% (5/8)	37.5% (3/8)
SCORE	Low	100%(8/8)	0%(0/8)
	Intermediate low and high	40%(8/20)	60%(12/20)
	High and very high	100%(1/1)	0%(0/1)
mSCORE	Low	100%(8/8)	0%(0/8)
	Intermediate low and high	40%(8/20)	60%(12/20)
	High and very high	100% (1/1)	0% (0/1)
mSCORE(BMI)	Low	80%(4/5)	20%(1/5)
	Intermediate low and high	62.5%(10/16)	37.5%(6/16)
	High and very high	37.5(3/8)	62.5%(5/8)
ARIC (m)	Low	56%(10/18)	44%(8/18)
	Intermediate low and high	64%(7/11)	36%(4/11)
	High and very high	0%(0)	0%(0)
PROCAM (m)	Low	63%(12/19)	37%(7/19)
	Intermediate low and high	57%(4/7)	43%(3/7)
	High and very high	33%(1/3)	67%(2/3)
Qrisk2	Low	71.5%(10/14)	28.5%(4/14)
	Intermediate low and high	37.5%(3/8)	62.5%(5/8)
	High and very high	57%(4/7)	43%(3/7)
QriskBMI	Low	73%(11/15)	27%(4/15)
	Intermediate low and high	29%(2/7)	71%(5/7)
	High and very high	57%(4/7)	43%(3/7)
Reynolds (m)	Low	62%(16/26)	38%(10/26)
	Intermediate low and high	33%(1/3)	67%(2/3)
	High and very high	0%(0)	0%(0)

The overall agreement single models was ICC of 0,504 (0,366-0,665 IC 95%), p<.001 but with the average ICC measures of 0,913 (0,852 - 0,952 IC 95%), p<.001. In the risk categories depicted in **Table 3** in concordance with the risk model specific classes (low, intermediary, high) Goodman–Kruskal γ test was used to measure the strength of association with the gold standard risk categories for each individual test. Out of the 9 tests, only two reached statistically significant strength (SCORE γ: 0,778, p=.005 and mSCORE γ:0,778, p=.005). Applying the *m* modifier led to no change in the classification in either of the risk categories.

The AUROCs for the prediction of subclinical atherosclerosis of each of the models are depicted in **Table 4** and showed wide variations in prediction capacity. The lowest ranking was ARIC, FRS, modified FRS, PROCAM, RRS, QRisk2 BMI with AUROCs inferior to.7. Three other will reach the .7 AUROC but will not reach the statistical significance (SCORE-BMI, mSCORE-BMI and Qrisk2). Only two scores reached the statistical significance: mSCORE (AUROC 0.775, IC95% 0,602-947, p=013) and SCORE (AUROC 0.792, IC95% 0,627-956, p=.008).

**Table 4:** AUROC for subclinical atherosclerosis

			95% IC	
Test Result	AUROC	P value	Lower Bound	Upper Bound
SCORE	,792	,008	,627	,956
FRS	,635	,223	,429	,841
mSCORE	,775	,013	,602	,947
mFRS	,635	,223	,430	,840
SCORE - BMI	,716	,051	,526	,906
mSCORE - BMI	,713	,054	,523	,904
RRS	,669	,127	,467	,871
PROCAM	,647	,184	,446	,848
ARIC	,537	,740	,323	,751
QRisk2	,701	,069	,510	,892
Qrisk2 - BMI	,679	,106	,484	,874

## Discussion

Predicting cardiovascular risk in rheumatoid arthritis proved to be a difficult task for the tested models. If the overall agreement between scores was good, most of the models tested in our study were not able to predict subclinical atherosclerosis as defined by the gold standard. Rheumatoid arthritis cardiovascular risk was higher than in the general population but also the risk profiles differed [**19**,**20**] and this was able to justify our findings. In our population RA was incompletely controlled by the ongoing treatment (high overall DAS 28) and inflammation was prevalent. ESR was found to be significantly higher in those patients with subclinical atherosclerosis at ultrasound. This was consistent with findings from previous studies where ESR was associated with a fourfold increase in CDV risk [**21**]. Also, the low total cholesterol and HDL cholesterol characterizing active RA (and other inflammatory profiles) would probably interfere with the results [**22**]. The presence of the rheumatoid factor also proved to increase the CVD risk by a twofold in a previous study where its prevalence was of 68% [**19**]. In our population, RF was present in 93.3% of the patients. The other particularities of this specific population were a high glucocorticoid and NSAID exposure. Methotrexate was inversely correlated with subclinical atherosclerosis, with a protective role in our study, consistent with the previous findings [**23**]. What was also noted was a prevalence of diabetes [**24**] higher than expected, a prevalence of smoking [**25**] lower than expected, but also a chronic kidney disease. A higher weight (and BMI), even if more prevalent in RA denoted a lower disease burden [**26**,**27**]. Our patients were discretely overweight but less than expected in RA [**27**,**28**]. These multiple interferences could explain the difficulties encountered by all the models tested in actually predicting subclinical atherosclerosis.

The only score reaching statistical significant prediction capacity in our RA population was the European SCORE model. SCORE well defined the low risk category with none of the patients classified in the low risk (below 1%), having either a plaque or a high IMT, but the overall SCORE underestimated the ultrasound predicated atherosclerosis risk. Only one patient reached the high-risk class out of the 12 classified as having subclinical atherosclerosis by ultrasound measurements. In fact, most of the patients found themselves in the intermediate risk class (20 out of 29). Interestingly, applying the EULAR modifier did not lead to any reclassification. It should be noted that the SCORE model used in our study follows the ESC recommendation and it is in conformity with the estimated risk in the Romanian general population.

All the office based (BMI) models were outperformed by their lipid based counterparts. This could be explained by the RA specific weight profile and also by a general loss of specificity by the surrogate marker (BMI for lipids). They failed to clearly identify either a low or high-risk populations. In the specific case of mSCORE-BMI, even though it had a lower AUC than its counterpart, the office-based model was a better predictor than all the other lipid- based models discussed.

Even though FRS is a consecrated risk prediction model, it only had a modest prediction capacity, even when the EULAR modifier was applied (mFRS). The other 2 models derived from the United States cohorts (ARIC, Reynolds) had similar results. When compared to SCORE, the three underestimated CVD risks, fail to clearly identify a low or high-risk category. PROCAM, the German derived model, also failed to do so. These results were also partially consistent with a recently published longitudinal study (Arts et al.) performed on a Netherlands rheumatoid arthritis population comparing SCORE, Reynolds FRS and Qrisk2 [**29**]. Failure to identify the high-risk category was also consistent with the Spanish cross-sectional study by Gómez-Vaquero et al., where even in a low-risk general population and by using scores specifically calibrated (REGICORE is the Spanish-calibrated variant of FRS and SCORE followed both ESC and EULAR recommendations), the high-risk population was under identified [**30**].

Qrisk2 is a model derived in UK that takes into consideration two possibly interesting aspects. It is calibrated from baseline to include RA as a risk factor among the other co morbidities like CKD, diabetes and hypertension and takes into account the local socioeconomically factor. In our population, Qrisk2 and Qrisk2 BMI underestimated risk and failed to identify a low-risk category. This result contravenes with the above mentioned study by Arts et al. where Qrisk2 overestimated overall CVD risk [**29**]. In our case, the specific genetic and socio-economical differences with the derivation population could explain some of the lack of specificity and sensitivity.

Our study had several limitations. It was a cross-sectional, small sample size study with the inherent limitations. The surrogate gold standard, even though validated and accepted does not imply a 100% specificity and sensitivity. The tertiary care recruitment site could explain a higher overall RA disease burden in our population. The “cardiovascular disease free” criteria might induce a selection bias especially in patients with longstanding RA. If generalized, the results of our study could only be used to characterize a Romanian population of RA, with the specific socio-economical and genetic background.

## Conclusion

Subclinical atherosclerosis and general cardiovascular risk prediction in rheumatoid arthritis by the available model are insufficient. The only model that provided usable information in a Romanian RA population was the SCORE model. Recalibration is needed. As recommended in the general population, the use of imaging diagnostic techniques in the intermediate risk population might ameliorate performance.

**Acknowledgments**

Funding: This work was funded by a grant of the Romanian Medical Academy, CEEX116/2006.
